# Discovery of New Secondary Metabolites by Epigenetic Regulation and NMR Comparison from the Plant Endophytic Fungus *Monosporascus eutypoides*

**DOI:** 10.3390/molecules25184192

**Published:** 2020-09-12

**Authors:** Zhe Guo, Zhong-Mei Zou

**Affiliations:** Institute of Medicinal Plant Development, Chinese Academy of Medical Sciences and Peking Union Medical College, Beijing 100193, China; guozhe0401@126.com

**Keywords:** epigenetic regulation, NMR comparison, *Monosporascus eutypoides*, cyclopropane derivatives, cytotoxic activities

## Abstract

Overexpression of the histone acetyltransferase and the ^1^H NMR spectroscopic experiments of the endophytic fungus *Monosporascus eutypoides* resulted in the isolation of two new compounds, monosporasols A (**1**) and B (**2**), and two known compounds, pestaloficin C (**3**) and arthrinone (**4**). Their planar structures and absolute configurations were determined by spectroscopic analysis including high resolution electrospray ionization mass spectroscopy (HRESIMS), one-dimensional (1D) and two-dimensional (2D) NMR, and calculated electronic circular dichroism data. Compounds **1–2** were screened in cytotoxic bioassays against HeLa, HCT-8, A549 and MCF-7 cells. Our work highlights the enormous potential of epigenetic manipulation along with the NMR comparison as an effective strategy for unlocking the chemical diversity encoded by fungal genomes.

## 1. Introduction

The epigenetic manipulation of fungal gene expression has proven to be an appropriate approach to unlock cryptic secondary metabolism pathways in several genera of filamentous fungi [1,2,3,4,5]. As one method of epigenetic manipulation, histone modifications can affect chromatin conformation and recruit proteins that cause epigenetic changes by interacting with histones [6]. As examples, the overexpression of the histone acetyltransferase in *Aspergillus nidulans* leads to the over production of sterigmatocystin, penicillin and terrequinone, particularly at earlier time points [7]. Cephalanones A–F, colletorin D, and colletorin D acid are unique metabolites produced by target strains after epigenetic manipulation [8,9]. Groot et al constructed the genetic transformation system of filamentous fungi in 1998 [10], the *Agrobacterium tumefaciens*-mediated transformation (ATMT) system has been a common technique for manipulation of gene expression in several fungi, and wildly used in the discovery of secondary metabolites [11,12,13,14].

At the same time, the special chemic shifts of the natural product can be an effective probe to finger out the unique structure fragments of the metabolites from the complicated secondary metabolites [15,16,17,18]. Guided by ^1^H NMR spectroscopic experiments, 11 macrocyclic diterpenes were isolated from the aerial parts of *Euphorbia helioscopia* using the aromatic protons as probes [17]. The assigned ^1^H and ^13^C chemical shifts leaded to the targeted isolation of a series of identified spiro compounds from *Carthamus oxyacantha* [18]. The structural motif-based approach “SUMMIT Motif” has also been proved efficient in the de novo identification of unknown molecular structures in complex mixtures [15].

Thus, the method of epigenetic regulation combining with NMR comparison could be an effective method in the discovery of natural products with unique structure motifs.

**Figure 1:**
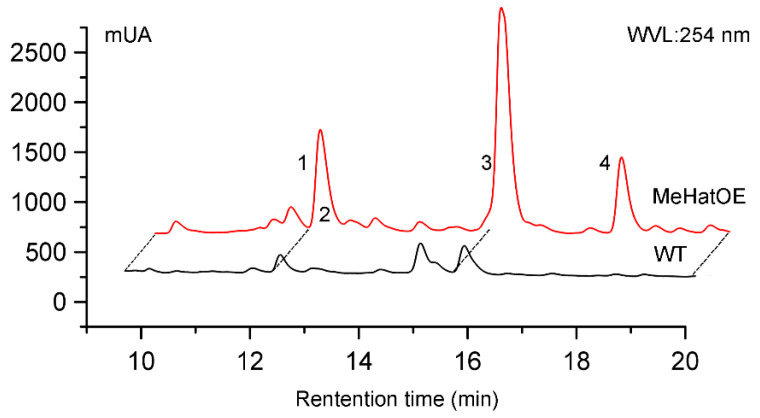
High-performance liquid chromatography (HPLC) chromatograms of the EtOAc extracts from the culture of WT (black) and MeHatOE (red), respectively (Chromatographic conditions: 0–2 min 60% MeOH in water, 2–25 min 60–100% MeOH/H_2_O, 25–30 min 100% MeOH, t = 30 min, 1.0 mL/min, 254 nm).

The endophytic fungus, *Monosporascus eutypoides,* was isolated from the root of the desert plant *Agriophyllum squarrosum* in Tengger desert of Ningxia province, People’s Republic of China. The genome of *M. eutypoides* revealed that this genome comprising 171 predicted SM key genes and 66 clusters (Figure S1). High-performance liquid chromatography (HPLC) analysis of crude extract of *M. eutypoides* grown in potato-dextrose broth (PDB) medium showed that only a few secondary metabolites were produced (Figure 1), implying that many of the gene clusters in this fungus were not, or only minimal, expressed. Through epigenetic manipulation, the production of the secondary metabolites can be improved, thus, the availability of molecular genetic tools to explore the new secondary metabolites is of big interest. However, to our knowledge, the applicability of ATMT in *M. eutypoides* has not been tested so far, the genetic transformation efficiency of different fungi is different, and the appropriate selection tags also need to be filtrated [19].

**Figure 2:**
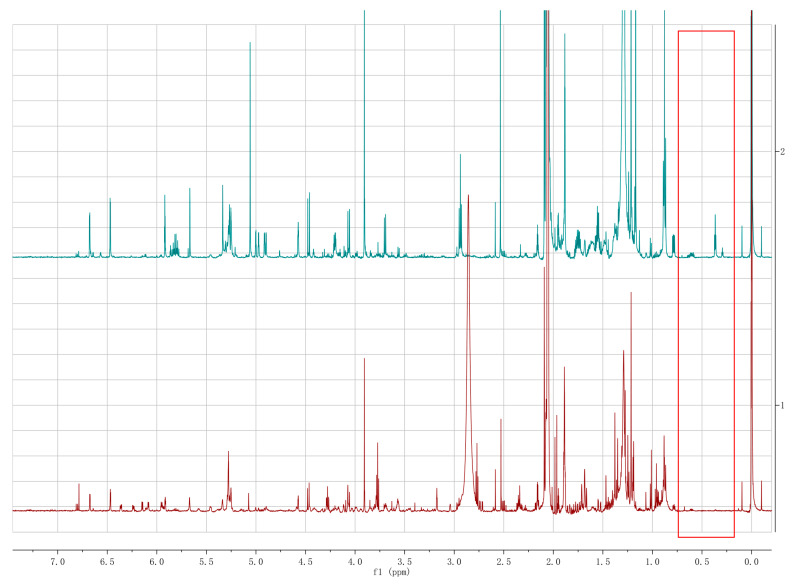
^1^H NMR spectra of the WT (brown) and MeHatOE (dark green), respectively (600 MHz, Acetone-*d*_6_).

To explore more secondary metabolites from *M. eutypoides*, we constructed the genetic transformation system of *M. eutypoides*, and compared the HPLC profile and ^1^H NMR data of the ethyl acetate (EtoAc ) extracts of the wild-type (WT) with those of the mutant strains (Figure 1 and Figure 2). The results showed that some new peaks were emerged (Figure 1) from the fermentation cultures of MeHatOE, which displayed characteristic cyclopropane proton signals in the *δ*_H_ 0.30−1.50 region in their ^1^H NMR spectra [20,21,22]. Therefore, these characteristic cyclopropane protons were employed as the probes for new compounds during our isolation procedure.

Guided by ^1^H NMR experiments and HPLC analysis, two new natural products containing unique cyclopropane ring, monosporasols A–B (**1**–**2**), together with the known pestaloficin C (**3**) and arthrinone (**4**) were isolated. Herein, the details of the establishment of genetic transformation system in *M. eutypoides*, ^1^H NMR comparison, isolation, structural elucidation, and biological evaluation of these compounds are presented.

## 2. Results

### 2.1. The ATMT Is an Effective Manipulation of Gene Expression in M. eutypoides

**Figure 3:**
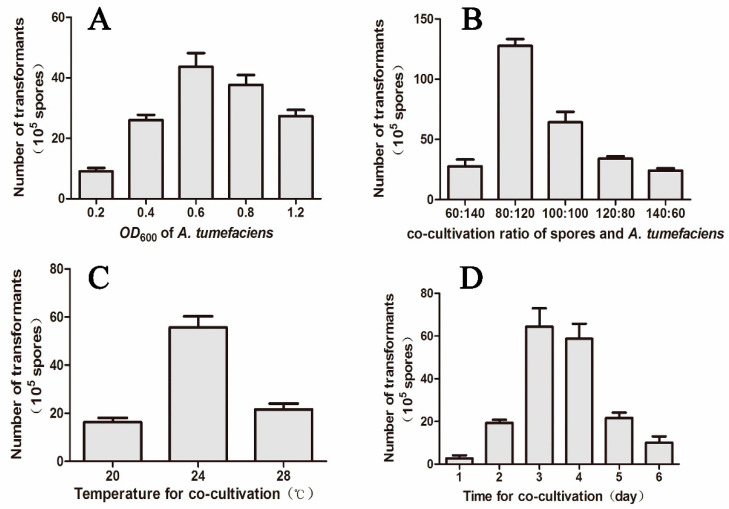
The parameters on the *Agrobacterium tumefaciens*-mediated transformation (ATMT) of *M. eutypoides*. (**A**) *OD_600_* of *A. tumefaciens*; (**B**) co-cultivation ratio of spores and *A. tumefaciens*; (**C**) temperature for co-cultivation; (**D**) time for co-cultivation. Bars denote standard error. Different letters in the same column in the same cultural condition indicate significant difference at *p* ≤ 0.05 level by the Tukey–Kramer multiple comparison test.

To construct the genetic transformation system of *M. eutypoides*, the level of sensitivity to hygromycin B (Figure S2) and the different conditions such as the *OD*_600_ of *A. tumefaciens*, the co-cultivation ratio of *A. tumefaciens* and spores, the co-cultivation times and the co-cultivation temperatures for the optimization of conditions were tested (Figure 3). Our results demonstrated that the transformation efficiency for *M. eutypoides* could reach at a high yield of 120–140 transformants per 10^6^ spores at optimized transformation conditions, including the 2:3 rate of *A. tumefaciens* (*OD*_600_ = 0.6) and the spore of *M. eutypoides* (10^7^ spores/mL), the co-cultivation temperature of 24 °C, the co-cultivation time of 3 days. The validity and sustainability of the optimized transformation condition was further verified by polymerase chain reaction (PCR), fluorescence observation and Western blot of *egfp* (enhanced green fluorescent protein) (Figure S2). Through the established ATMT protocol we proved that the *hyg* gene could be used as a selective marker and *egfp* as a reporter gene for expressing the fluorescent reporter genes in the wild-type fungus or mutants to visualize the host colonization. The results confirmed that ATMT was an efficient means of producing insertional mutagenesis and subsequent identification of mutated genes in *M. eutypoides*, which should facilitate further genetic manipulation in this fungus.

### 2.2. Identification of MeHat and the Overexpression of MeHat in M. eutypoides

The *Mehat* gene (GenBank accession number MK 590051) was cloned from *M. eutypoides*. The ORF was 1627 bp in size, which encoded a 502 amino acid polypeptide. The molecular weight of the protein was 64.2 kDa. Sequence analysis showed that there was a conserved histone acetyltransferase binding domain in the N-terminal region of the 5rd–163rd amino acids (Figure S3).

**Figure 4:**
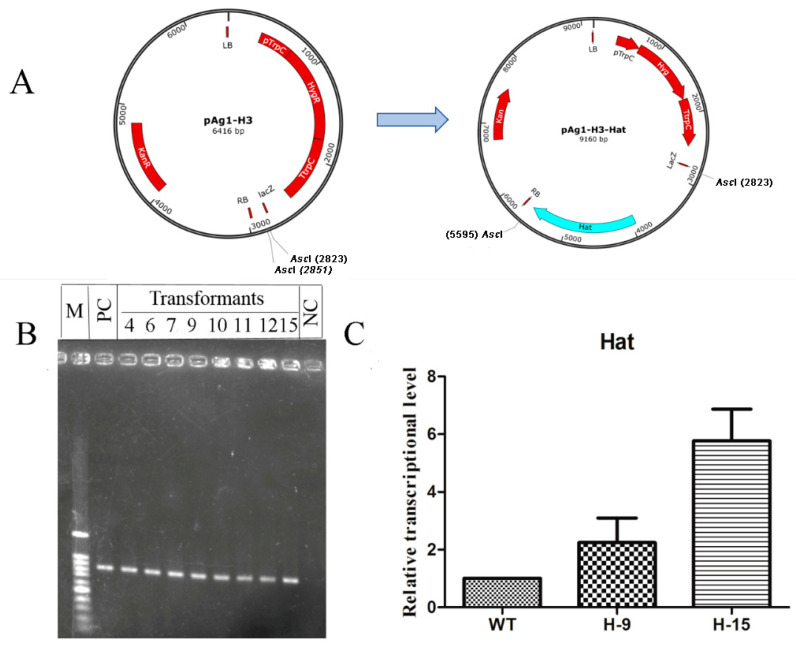
(**A**) Schematic diagram of the *hat* gene overexpression strategy. The *hat* promoter and *hat* gene were cloned in the pAg1-H3 vector to generate overexpression vector pAg1-H3-Hat. (**B**) PCR was performed to select MeHatOE. Hyg-F and Hyg-R were PCR primers. PC: positive control; NC: negative control; M: 100 bp ladder. (**C**) RT-PCR was used to detect the transcription of *hat* in MeHatOE relative to wild-type (H-9 and H-15 were randomly selected for RT-PCR experiments).

The MehatOE cassette was amplified to construct a *Mehat* overexpression plasmid (Figure 4A), then the MehatOE plasmid was transformed to *M. eutypoides* via the ATMT method and screened with 80 μg/mL hygromycin B in PDA media. Eight MehatOE mutants were obtained and confirmed by PCR (Figure 4B). Subsequently, two strains (H-9 and H-15) were selected randomly for real-time PCR (RT-PCR). The *Mehat* expression levels of H-9 and H-15 mutants were, respectively, three-fold and six-fold higher than that in the WT (Figure 4C).

### 2.3. Secondary Metabolite Assessment by HPLC Chromatograms and ^1^H NMR Analysis

Due to the higher expression of *Mehat*, H15 was selected as MeHatOE for the further experiment. MeHatOE was cultivated in PDB for 7 days at 28 °C and 150 rpm. The culture medium (1 L) was extracted thrice with ethyl acetate. The wild-type was fermented in the same condition. The comparison of the HPLC chromatograms and ^1^H NMR of the MehatOE (Figure 1 and Figure 2) and the wild-type extracts showed that the compounds with unique cyclopropane ring produced in mutant were either not detectable or produced in only small amounts by the wild-type (Figure 1). In order to figure out the compounds with unique cyclopropane ring, the ethyl acetate extract of MehatOE was separated by silica gel column chromatography, and reversed-phase HPLC to afford monosporasols A (**1**) and B (**2**), pestaloficin C (**3**) and arthrinone (**4**). All these compounds (Figure S5) were analyzed using the standard method (0–2 min 60% MeOH in water, 2–25 min 60–100% MeOH/H_2_O, 25–30 min 100% MeOH). All these new compounds were identified by mass spectrometry, 1D and 2D NMR, and experimental electronic circular dichroism (ECD).

Monosporasol A (**1**) was obtained as yellow oil and was assigned the molecular formula as C_16_H_20_O_4_ (seven degrees of unsaturation) on the basis of its high resolution electrospray ionization mass spectroscopy (HRESIMS) and NMR data (Table 1). Analysis of its NMR data revealed the presence of two exchangeable protons (*δ*_H_ 4.26 and 4.32, respectively), three methyl groups, two methylene units, seven methines, two olefinic carbons (one of which was protoned), two alkyne carbons (*δ*_C_ 86.7 and 92.7, respectively), and three sp^3^ quaternary carbons (one of which was oxygenated). These data accounted for all the resonances observed in ^1^H and ^13^C NMR spectra of **1**.

**Figure 5:**
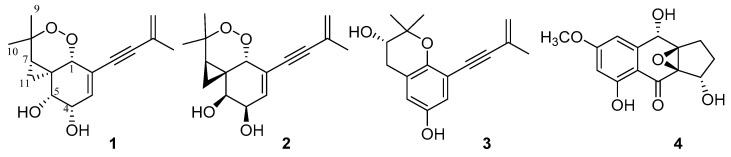
Structures of **1**−**4** from MeHatOE.

**Figure 6:**
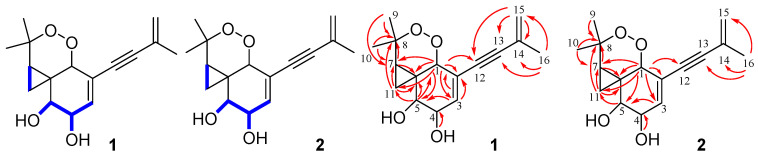
Key ^1^H-^1^H COSY (blue lines) and HMBC (red arrows) correlations of compounds **1** and **2.**

Interpretation of ^1^H–^1^H COSY NMR data of **1** showed two isolated proton spin-systems of C-3–C-5 (including OH-4 and OH-5) and C-7–C-11. Heteronuclear multiple bond correlation (HMBC) correlations from H-5 to C-1 and C-6, whereas those correlations from H-1 to C-2, C-3, C-5, C-6 and C-12, and H-3 to C-1, and C-2 established a penta-substituted cyclohexene ring with a ethyne substituted at C-2. In turn, correlations in the HMBC spectrum from H-5 to C-6, and C-11 revealed the presence of a cyclopropane which was spirally jointed to the cyclohexene moiety at C-6. Additional correlations from H_3_-9 and H_3_-10 to C-7 and C-8 located both methyls at C-8 and attached the sp^3^ quaternary carbon to C-7. Furthermore, considering the chemical shift of carbons C-1 (*δ*_C_ 76.8) and C-8 (*δ*_C_ 82.6), as well as the unsaturation requirement of **1**, the two carbons were connected via a peroxide bridge to complete the planar structure of **1,** as shown in Figure 5. Finally, HMBC correlations (Figure 6) from H_3_-16 to C-14, and C-15, and H_2_-15 to C-13, and C-14 attached the methyl and ethyne groups to olefin subunit at C-14, leading to the assignment of the planar structure of monosporasol A, as shown in Figure 5.

**Figure 7:**
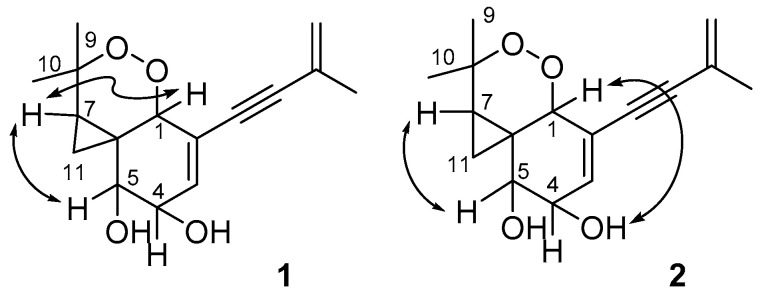
Key NOESY correlations of compounds **1**–**2.**

The relative configuration of **1** was also assigned by analysis of ^1^H–^1^H coupling constants and nuclear overhauser effect spectroscopy (NOESY) data (Figure 7). The small coupling constants of H-4/H-5 (7.2 Hz) observed demonstrated a *syn*-relative orientation of the two protons. While correlations in the NOESY spectrum of H-7 with H-1 and H-5 showed the special proximity of these protons.

**Figure 8:**
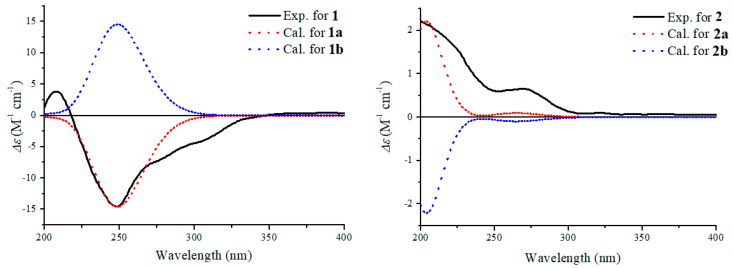
Experimental electronic circular dichroism (ECD) spectrum of **1**–**2** in MeOH and the calculated circular dichroism (CD) spectra of **1a**–**2a** and **1b**–**2b**.

The absolute configuration of **1** was proposed by comparison of the experimental and simulated electronic circular dichroism (ECD) spectra calculated using the time-dependent density functional theory (TDDFT) [20,21,22,23,24,25] for two isomers, (1*R*, 4*S*, 5*R*, 6*R*, 7*S*)-**1** (**1a**) and (1*S*, 4*R*, 5*S*, 6*S*, 7*R*)-**1** (**1b**). A systematic conformational analysis was performed for **1a** and **1b** with the molecular operating environment (MOE) software package using the MMFF94 molecular mechanics force field calculation. The selected conformers were then reoptimized using TDDFT at the CAM-B3LYP/6-31++G(2d,2p) level with the Conductor-like Polarized Continuum Model (CPCM) to afford the lowest energy conformers. The overall calculated circular dichroism (CD) spectra of **1a** and **1b** were then generated by Gaussian broadening. The experimental CD curve of **1** was nearly identical to that calculated for **1a** (Figure 8), suggesting the 1*R*, 4*S*, 5*R*, 6*R*, 7*S* absolute configuration for **1**.

The molecular formula of monosporasol B (**2**) was determined to be C_16_H_20_O_4_ (seven degrees of unsaturation) on the basis of HRESIMS data, which was identical to that of compound **1**. Analysis of its NMR data (Table 1) revealed the same planar structure as **1**.

The small coupling constant of 5.4 Hz between H-4 and H-5 lead to a *syn*-relative orientation of the two protons. The correlation between H-5 and H-7 showed them to be cofacial, while a correlation of H-1 and 4-OH showed H-1, 4-OH and 5-OH were on the opposite of the ring system. Therefore, the relative configuration of **2** was proposed as shown. To determine the absolute configuration in **2,** the experimental CD spectra was compared with the calculated CD spectra of the enantiomers (1*R*, 4*R*, 5*S*, 6*S*, 7*R*)-**2** (**2a**) and (1*S*, 4*S*, 5*R*, 6*R*, 7*S*)-**2** (**2b**). The experimental CD spectrum of **2** matched only the calculated CD curve of **2a** (Figure 8). Therefore, the absolute configuration of **2** was assigned 1*R*, 4*R*, 5*S*, 6*S*, 7*R*.

Besides the new monosporasols A–B (**1**–**2**), the known pestaloficin C (**3**) [14] and arthrinone (**4**) [26] were also isolated from MeHatOE by interpretation of their spectroscopic data as well as comparison with those reported in the literatures.

Compounds **1**–**2** were evaluated for their cytotoxicity against HeLa (cervical cancer cells), HCT-8 (human colon adenocarcinoma cells), A549 (human lung adenocarcinoma cells), and MCF-7 (human breast cancer cells) using the 3-(4,5-dimethylthiazol-2-yl)-2,5-diphenyltetrazolium bromide (MTT) method. Unfortunately, none of them had marked cytotoxicity between 5 µM to 50 µM (Table S3).

## 3. Discussion

The endophytic fungus *M. eutypoides* was isolated from the root of the desert plant *A. squarrosum***,** the unique habitat, and the endophytic fungi were recognized as an important source of structurally diverse and pharmacologically active natural products [27,28,29,30,31,32,33]. In our previous study, the wild-type showed little production in the secondary metabolites, considering the genetic manipulation is a common technique for the discovery of new products [34], the availability of molecular genetic tools to explore its biology and secondary metabolites is of big interest.

ATMT has become a common technique for selected fungi including endophytes [11]. Like other fungi, the conversion efficiency of *M. eutypoides* is affected by many factors. The transformation frequency largely depends on the amount of *A. tumefaciens* cells or fungal recipient cells in the co-cultivation mixture, the higher mount, and the larger frequency [35], however, there is a limitation in the concentration of the both components, and too high or too low proportion of them can decrease the transformation efficiency dramatically [35,36]. The time of the co-cultivation also affects the transformation efficiency, and too long co-cultivation periods could decrease the transformation efficiency and the excess growth of the fungus could lead to a false positive [37,38,39]. In this study, the ATMT system for *M. eutypoides* had been established for the first time.

Since the modulation of histone acetylation affects gene expression [7,40], the histone acetylase gene, *MeHat,* was overexpressed by the ATMT in *M. eutypoides*. The HPLC analysis was compared between the fermentation cultures of MeHatOE and wild-type, some new peaks were emerged from MeHatOE, which displayed characteristic cyclopropane proton signals in the *δ*_H_ 0.3−1.50 region in their ^1^H NMR spectra, therefore, the isolation guided by the ^1^H NMR lead to the discovery of two new natural products containing unique cyclopropane ring, monosporasols A and B (**1**–**2**).

Cyclopropane subunits occur in many natural products, many of them show biological activity and may serve as potential drug leads [41]. Monosporasols A and B are new additions, and the isolation of compounds **1** and **2**, guided by epigenetic regulation and NMR comparison may provide a new pattern for the discovery of novel natural products.

## 4. Materials and Methods

### 4.1. General Experimental Procedure

Optical rotations were measured on a Rudolph Research Analytical automatic polarimeter, and UV data were obtained on a Shimadzu Biospec-1601 spectrophotometer (Shimadzu, Kyoto City, Kyoto Prefecture, Japan). CD spectra were recorded on a JASCO J-815 spectropolarimeter (Jasco, Essex, UK). IR data were recorded using a Nicolet Mag 750 spectrophotometer (Waltham, MA, USA). ^1^H and ^13^C NMR data were acquired with Bruker Avance III-600 spectrometer (Burker, Billerica, MA, USA) using solvent residual signals (Acetone-*d*_6_: *δ*_H_ 2.05/*δ*_C_ 29.8, 206.1) as references. The heteronuclear single quantum coherence (HSQC) and HMBC experiments were optimized for 145.0 and 8.0 Hz, respectively. Electrospray ionization tandem mass spectrometry (ESIMS) and HRESIMS data were obtained using an Agilent Accurate-Mass-Q-TOF LC/MS G6550 instrument equipped with an electrospray ionization (ESI) source (Agilent, Santa Clara, CA, USA). All MS experiments were performed in the positive ion mode. Semi-preparative HPLC separation was performed on a Shimadzu LC-6AD instrument packed with a YMC-Pack ODS-A column (5 µm, 250 mm × 10 mm) (Shimadzu, Kyoto City, Kyoto Prefecture, Japan). Sephadex LH-20 was purchased from Pharmacia Biotech (Uppsala, Sweden). Silica gel (200–300 mesh) for column chromatography was produced by Qingdao Marine Chemical Factory, Qingdao, China.

### 4.2. Biology Materials

The culture of *M. eutypoides* was isolated from the root of the desert plant *A. squarrosum* and was identified based on morphology and sequence (Genebank Accession No. KT347319) analysis of the ITS (Internal Transcribed Spacers) region of the rDNA (Ribosomal DNA). The culture of the strain was deposited in China General Microbiological Culture Collection Center at the Institute of Microbiology, the Chinese Academy of Sciences with the accession No. 3.17736. *Escherichia coli* DH5α (TranGene, Beijing, China) was used as a host for all DNA manipulations. *E.coli* was routinely grown at 37 °C in Luria-Bertani(LB) medium [42] with antibiotics when required. For sporulation, *M. eutypoides*, MeHatOE9 and MeHatOE15 were incubated in potato dextrose agar (PDA; 200 g/L potato, 20 g/L glucose, and 20 g/L agar) at 28 °C for 5 days. Modified mediums such as minimal medium (MM), induction medium (IM) and co-cultivation medium(CM) for ATMT were prepared as previously described [43]. Dulbecco’s Modified Eagle Medium (DMEM medium, Gibco Company, New York, NY, USA) supplemented with 10% fetal bovine serum (FBS, PAN Company, Aidenbach, Germany) was used for MTT assay. PDA supplemented with 80 μg/mL hygromycin B (Amresco, Solon, OH, USA) were used to screen hygromycin-resistant transformants. All cultures of fungi were grown at 28 °C in an incubator. *A. tumefaciens* strain AGL-1 was used as T-DNA(Transfer DNA)donor for fungal transformation of *M. eutypoides*. pAg1-H3-EGFP plasmid, carrying hygromycin B resistance gene and green *egfp*, was kindly provided by Professor Gang Liu (Institute of Microbiology, Chinese Academy of Sciences).

The bacterial and spore counts were calculated by hemocytometer.

All of the primers used in this study are listed in the Table S1. DNA extraction, PCR analysis were performed as described previously [44].

The total RNA was extracted with NucleoSpin RNA (Macherey-Nagel, Dylan, Germany) according to the manufacturer’s protocol. RT-PCR analysis was carried out as described previously [45].

The SDS-PAGE, Western blot, immunoblot and fluorescence microscopic observation were evaluated as in Reference [46]. The EGFP antibody (Abmart, Cambridge, MA, USA) was diluted in a scale of 1:1000 and ant-mouse horseradish peroxidase-conjugated IgG (Abmart, Cambridge, MA, USA) was used as the secondary antibody at a 1:4000 dilution.

### 4.3. Construction of ATMT System for M. eutypoides

To construct the ATMT of *M. eutypoides*, the sensitivity of *M. eutypoides* to hygromycin B was tested using increasing concentrations of hygromycin B antibiotic compared to a control plate without hygromycin B (Figure S2). Then, the ATMT was carried out as described [43] with some suitable adjustments. The bacterial strain was grown in MM medium at 28 °C, 200 rpm until the culture reached an *OD*_600_ value of 0.8–0.9, then the agrobacterium cells were diluted to *OD*_600_ = 0.15–0.2 in IM amended with kanamycin and 200 μM acetosyringone (AS) and incubated for an additional 6 h at 28 °C at 250 rpm (*OD*_600_ reached around 0.6). The spores of *M. eutypoides* were collected from 5–7 days old cultures grown on PDA, then filtered the culture through two layers of miracloth to remove mycelia and suspended in water and their concentration adjusted to 10^7^ spores/mL. The spore suspension was mixed with *A.tumefaciens* culture in a series of proportions (40:160, 80:120, 100:100, 120:80 and 160:40), a 200 μL of each ratio of mixture was spread on a nitrocellulose membrane placed on CM in a small plate of IM containing with 200 μM AS. The co-culture step was carried out in the dark with different time intervals (2, 3, 4 days). After co-cultivation growth, the membranes/papers were transferred to PDA plates amended with 200 μM cefotaxime (to kill *A. tumefaciens*) and 80 μg/mL hygromycin B for selecting fungal transformants. Putative transformants were transferred to PDA plate supplemented with 80 μg/mL hygromycin B. The plates were incubated at 25 °C for 4–5 days to obtain fungal transformants. The transformation efficiency of the ATMT method for the *M. eutypoides* strain should be tested for different conditions such as the *OD*_600_ of *A. tumefaciens*, the co-cultivation ratio of *A. tumefaciens* and spores, the co-cultivation times and temperature for the optimization of conditions, these conditions were experimented in triplicate. The efficiency of the ATMT method was tested by Western blot analysis and fluorescence microscopy of *egfp*, which was integrated in *M. eutypoides* by the optimistic condition of ATMT [46].

### 4.4. Construction of the MeHatOE

The *MeHat* gene was acquired from the database of genomic sequencing of *M. eutypoides*. The base sequence of *MeHat* gene was analyzed by an online BLAST search at the National Center for Biotechnology Information (NCBI) website (http://www.ncbi.nlm.nih.gov/). The *MeHat* gene with its promoter region were PCR amplified from *M. eutypoides* using the Hat-F/Hat-R primers, and the amplified 2736 bp DNA fragment was ligated into pEASY-Blunt (TransGene, Beijing, China) to give pEASY-Hat. After verified by sequencing, pEASY-Hat was digested with *Asc*I, and the digested DNA fragment containing *hat* was ligated into the corresponding site of pAg1-H3 to give pAg-Hat (Figure 4A). Subsequently, the resulting pAg-Hat was introduced into *M. eutypoides* by ATMT with the conditions explored above.

After co-incubation on PDA at 28 °C for 3 days, the transformants were transferred to the PDA plates supplemented with 80 µg/mL hygromycin B and 400 µg/mL cefotaxime, and the hygromycin B resistant strains were selected. The overexpress strain (H-9 and H-15) was further verified by PCR with the RTHat-F/RTHat-R primers and the higher expression strain was selected for the further experiments.

### 4.5. Fermentation and Isolation

The WT and MeHatOE strain were grown on PDA plates at 28 °C for 7 days. The spores were filtered by two layers of miracloth to remove mycelia and suspended in water and their concentration adjusted to 10^7^ spores/mL, a total of five Erlenmeyer flasks of 500 mL each containing 200 mL of PDB medium were inoculated, and the contents were allowed to grow at 28 °C with shaking (150 rpm) for 7 days. The culture was soaked at room temperature with the recycled ethyl acetate many times until the extract was almost colorless. The solvent was removed under reduced pressure using a rotatory evaporator to afford a crude residue (1.3 g). The original extract was fractionated on a silica gel column using petroleum ether (PE)-Acetone (20:1–1:1) progressively to give 10 fractions (Fr.1 to Fr.10). Fr.4 (188.0 mg) was subjected to Sephadex LH-20 (CH_2_Cl_2_-MeOH, 1:1, *v/v*) to get three subfractions (Fr.4.1–Fr.4.3). Fr.4.1 (52.4 mg) was separated by semipreparative HPLC (60–65%MeOH in H_2_O for 20 min) to obtain monosporasol A (**1**, 6.0 mg, *t*_R_ 17.30) and pestaloficin C (**3**, 5.1 mg, *t*_R_ 19.30). Fr.4.2 (35.6 mg) was purified by semipreparative HPLC (55–62% MeOH in H_2_O for 35 min) to obtain monosporasol B (**2**; 0.9 mg, *t*_R_ 21.05 min). Fr.5 (135.6 mg) was subjected to separation by semipreparative HPLC (MeOH-H_2_O, 52% for 30 min) to obtain arthrinone (**4**, 2.1 mg, *t*_R_ 24.55).

Monosporasol A (**1**): Yellow oil (acetone); [α]^25^_D_ − 11.40 (*c* = 0.5, MeOH); UV (MeOH) *λ*_max_ (log *ε*) 291(4.10) nm; IR (Neat) *v*_max_: 3390, 2930, 1665 cm^-1^; HRESIMS *m*/*z*: 299.1270 (M + Na)^+^ (calcd 299.1254 for C_16_H_20_O_4_Na). ^1^H NMR (600 MHz, Acetone-*d*_6_) and ^13^C NMR (150 MHz, Acetone-*d*_6_) data, see Table 1.

Monosporasol B (**2**): Yellow oil (acetone); [α]^25^_D_ − 4.32 (*c* = 0.1, MeOH); UV (MeOH) *λ*_max_ (log *ε*) 292 (3.76) nm; IR (Neat) *v*_max_: 3379, 1685, 1366 cm^-1^; HRESIMS *m*/*z*: 299.1284 (M + Na)^+^ (calcd 299.1254 for C_16_H_20_O_4_Na). ^1^H NMR (600 MHz, Acetone-*d*_6_) and ^13^C NMR (150 MHz, Acetone-*d*_6_) data, see Table 1.

### 4.6. Computational Details

Conformational analysis within an energy window of 3.0 kcal/mol was performed by using the optimized potentials for liquid simulations (OPLS3) molecular mechanics force field via the Macro Model [47] panel of Maestro 10.2. The conformers were then further optimized with the software package Gaussian 09 [48] at the B3LYP/6-311G(2d,p) level, and the harmonic vibrational frequencies were also calculated to confirm their stability. Then the 60 lowest electronic transitions for the obtained conformers in vacuum were calculated using time-dependent density functional theory (TD-DFT) methods at the CAM-B3LYP/6-311G(2d,p) level. ECD spectra of the conformers were simulated using a Gaussian function with a half-bandwidth of 0.16 eV and 0.39 eV for compounds **1** and **2**. The overall theoretical ECD spectra were obtained according to the Boltzmann weighting of each conformers.

### 4.7. MTT Assay 

The anti-proliferative activity of the isolated new compounds against Hela, HCT-8, A549, and MCF-7 cell lines were evaluated by MTT method [49]. Target tumor cell lines were grown to the log phase in DMEM medium supplemented with 10% FBS at 37 °C under a 5% CO_2_ atmosphere. The cell suspension was added to 96 well plates at the density of 8 × 10^4^ cells/mL. After overnight incubation, test compounds were added. The plates were allowed to incubate for 48 h, and then 3-(4, 5-dimethylthiazol-2-yl)-2, 5-diphenyl tetrazolium bromide (MTT) was added to each well, and the optical density was measured at the 492 nm wavelength on Tecan Infinite M1000 microplate reader. Three replicate wells were used for each drug concentration in all experiments.

## 5. Conclusions

In working with the discovery of natural products, several research approaches have been reported to be involved in both secondary metabolism and fungal development [50,51]. It is urgent to find new approaches to distinguish new products from the known compounds. In this study, the optimal condition for the ATMT of *M. eutypoides* was established. Two new natural products containing unique cyclopropane rings, monosporasols A (**1**) and B (**2**) were isolated from the MeHatOE, guided by ^1^H NMR experiments and HPLC analysis. Our work emphasized on the effectiveness of combining genetic manipulation with NMR to discover novel natural products from *M. eutypoides*, which may facilitate further studies in fungi.

## Figures and Tables

**Table 1:** NMR Data for **1** and **2.**

Pos.	1			2		
	*δ*_C_*^a^*,mult.	*δ*_H_*^b^*(*J* in Hz)	HMBC	*δ*_C_*^a^*,mult.	*δ*_H_*^b^*(*J* in Hz)	HMBC
1	76.8, CH	4.41, dd (3.0, 2.4)	2, 3, 4, 5, 6, 7, 11, 12	77.3, CH	4.19, dd (2.4,1.8)	2, 3, 4, 5, 7, 11, 12
2	124.1, qC			128.8, qC		
3	139.5, CH	5.85, dd (3.0, 2.4)	1, 2, 5, 6	135.0, CH	6.11, ddd (5.4, 2.4, 1.2)	5
4	77.0, CH	4.15, m		69.1, CH	4.12, m	
5	72.7, CH	4.10, dd (7.2, 3.0)	1, 6, 11	75.0, CH	3.48, ddd (5.4, 3.6, 1.8)
6	36.5, qC			32.4, qC		
7	29.1, CH	1.64, dd (7.8, 3.0)	1, 5, 6, 8, 9, 10, 11	33.2, CH	1.64, dd (7.8, 4.2)	1, 5, 6, 8, 9
8	82.6, qC			82.3, qC		
9	24.8, CH_3_	1.23, s	7, 8, 10	28.4, CH_3_	1.33, s	
10	24.7, CH_3_	1.17, s	7, 8, 9	26.2, CH_3_	1.12, s	
11	3.9, CH_2_	0.60, dd (7.8, 3.6)	1, 5, 6, 7, 8	12.0, CH_2_	0.64, dd (7.8, 4.8)	1, 5, 6, 7, 8
		0.28, dd (3.6, 3.0)	1, 5, 6, 7, 8, 9, 10		0.58, dd (4.8, 4.2)	1, 5, 6, 7, 8
12	86.7, qC			89.0, qC		
13	92.7, qC			91.4, qC		
14	128.0, qC			128.0, qC		
15	122.0, CH_2_	5.27, m; 5.24, m	13, 14	121.8, CH_2_	5.26, m; 5.24, m	
16	23.6, CH_3_	1.87, br. s	13, 14, 15	23.6, CH_3_	1.88, br. s	13, 14, 15
4-OH		4.26, d (6.0)			4.41, d (4.2)	3, 4, 5,
5-OH		4.32, d (3.0)			4.42, d (3.6)	4, 5, 6

*^a^* Recorded at 150 MHz in Acetone-*d*_6_. *^b^* Recorded at 600 MHz in Acetone-*d*_6_.

## Supplementary Materials

The supplementary materials are available online.
