# Supplementary material for: Discovery of New Secondary Metabolites by Epigenetic Regulation and NMR Comparison from the Plant Endophytic Fungus Monosporascus eutypoides
Source: Molecules. 2020 Sep 12;25(18):4192. doi: 10.3390/molecules25184192 (PMC7570479; doi:10.3390/molecules25184192)
Supplement: Supplementary file 1 [file molecules-25-04192-s001.pdf]

Supplementary

Discovery of New Secondary Metabolites by Epigenetic Regulation and NMR Comparison from the Plant Endophytic Fungus *Monosporascus eutypoides*

Zhe Guo and Zhong-Mei Zou *

Institute of Medicinal Plant Development, Chinese Academy of Medical Sciences and Peking Union Medical College, Beijing 100193, China; guozhe0401@126.com

* Correspondence: zmzou@implad.ac.cn; Tel.: +86-10-5783-3290

Received: 14 August 2020; Accepted: 10 September 2020; Published: 11 September 2020

Supplementary Tables

Table S1. Primers used in this study..3
Table S2. Strains and plasmids used in this study...3
Table S3. Cytotoxicity of monosporasols A(**1**) and B (**2**)..4

Supplementary Figures

Figure S1. AntiSMASH analyzing the genome of *M. eutypoides*...5
Figure S2. The sensitivity of the *M. eutypoides* strain to different concentrations of hygromycin B. 5
Figure S3. PCR, fluorescence microscopy observation and Western blot of the transformants of *M. eutypoides*...6
Figure S4. Sequence alignment of Mehat protein...7
Figure S5. HPLC analysis of all these compounds with standard method..8
Figure S6. IR spectrum of compound **1**...9
Figure S7. HRESIMS spectrum of compound **1**..9
Figure S8. ^{1}H NMR spectrum of compound **1** (600 MHz, Acetone-d_6) 10
Figure S9. ^{13}C NMR spectrum of compound **1** (150 MHz, Acetone- d_6)..10
Figure S10. HSQC spectrum of compound **1** (600 MHz, Acetone- d_6)..11
Figure S11. ^{1}H-^{1}H COSY spectrum of compound **1** (600 MHz, Acetone- d_6)......................................11
Figure S12. HMBC spectrum of compound **1** (600 MHz, Acetone- d_6) 12
Figure S13. NOESY spectrum of compound **1** (600 MHz, Acetone- d_6)..12
Figure S14. IR spectrum of compound **2**..13
Figure S15. HRESIMS spectrum of compound **2**..13
Figure S16. ^{1}H NMR spectrum of compound **2** (600 MHz, Acetone- d_6)...14
Figure S17. ^{13}C NMR spectrum of compound **2** (150 MHz, Acetone- d_6)...14
Figure S18. HSQC spectrum of compound **2** (600 MHz, Acetone- d_6)..15
Figure S19. ^{1}H-^{1}H COSY spectrum of compound **2** (600 MHz, Acetone- d_6)......................................15
Figure S20. HMBC spectrum of compound **2** (600 MHz, Acetone- d_6) 16
Figure S21. NOESY spectrum of compound **2** (600 MHz, Acetone- d_6)..16
Figure S22. Five Conformations of **1a** were obtained after the Optimization....................................17
Figure S23. Twenty Conformations of **2a** were obtained after the Optimization. 17

Table S1. Primers used in this study

Primers	Sequence(5′-3′)
Primers used for constructing *MehatOE* mutant	
Hat-OE-F	GGCGCGCCAAATTTTTTCGCTCGGCG
Hat-OE-R	GGCGCGCCTTAAGTATCCTCGATTCGGATCT
Primers used for identifying *egfp* mutant	
GFP-F	GGCGCGCCATGAGTAAAGGAGAAG
GFP-R	GGACGTCCTTATTTGTATAGTTCATCCATGCC
Primers used for quantitative real-time PCR	
tubulin-RT-F	TCTTCCTCGTCAACACCA
tubulin-RT-R	ACCTTCGTCGGTAACTCG
Hat-RT-F	TCGCGTCCTTCCATCCTAAG
Hat-RT-R	CATAACCGCCGACACATCTG

Table S2. **Strains and plasmids used in this study**

**Strains/plasmids**	**Characteristics**	**Source**
**Strains**		
Escherichia coli DH5a	Strain used for routine cloning	Gibco BRL
Agrobacterium tumefaciens AGL-1	Strain used for fungal transformation	Khang et al.,2006 [1]
M. eutypoides	The wild-type strain	This study
Mehat	The *hat* overexpressed strain	This study
H-9	The *hat* overexpressed strain-9	This study
H-15	The *hat* overexpressed strain-15	This study
**Plasmid**		
pEASY-Blunt	Routine cloning vector	Transgen
pEASY-hat	The DNA fragment containing the downstream flanking sequence of *hat* was inserted into pEASY-Blunt	This study
pAg1-H3	The vector used for ATMT	Khang et al.,2006 [1]
pAg-egfp	The *egfp* gene was insert into pAg1-H3	Wang, et al..,2013 [2]
pAg-hat	The plasmid containing the entire *hat* used for the overexpression experiment	This study

Table S3. Cytotoxicity of monosporasols A(1) and B (2)

compound	IC_{50}(μM)			
	HeLa	HCT-8	A549	MCF-7
**1**	> 50	> 50	> 50	> 50
**2**	> 50	> 50	> 50	> 50
cispatin	14.13 ± 2.01	9.48±1.83	17.98 ± 2.77	18.15 ± 1.37

Figure S1. AntiSMASH analyzing the genome of *Monosporascus eutypoides*.

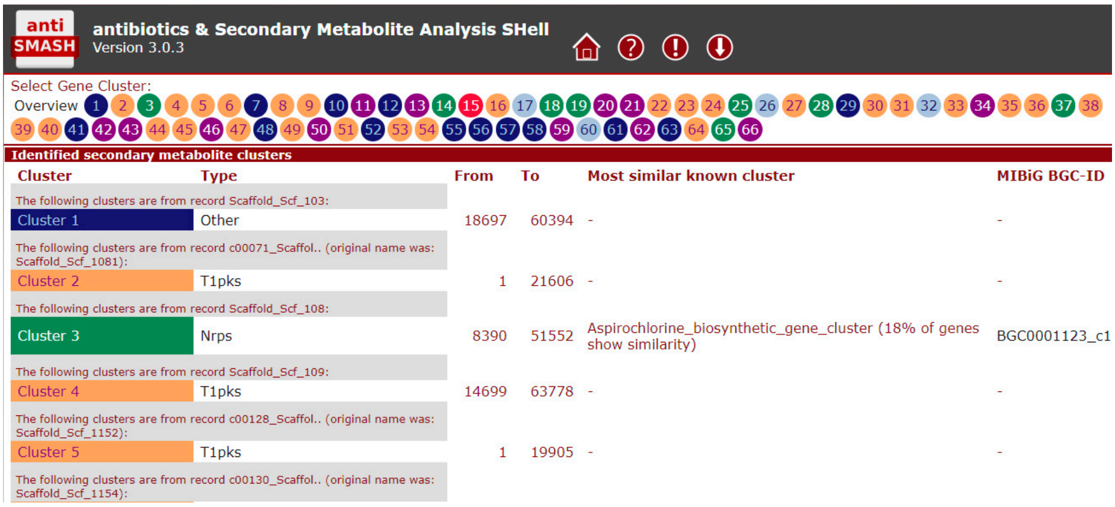

Cluster	Type	From	To	Most similar known cluster	MIBiG BGC-ID
The following clusters are from record Scaffold_Scf_103:					
Cluster 1	Other	18697	60394	-	-
The following clusters are from record c00071_Scaffol.. (original name was: Scaffold_Scf_1081):					
Cluster 2	T1pks	1	21606	-	-
The following clusters are from record Scaffold_Scf_108:					
Cluster 3	Nrps	8390	51552	Aspirochlorine_biosynthetic_gene_cluster (18% of genes show similarity)	BGC0001123_c1
The following clusters are from record Scaffold_Scf_109:					
Cluster 4	T1pks	14699	63778	-	-
The following clusters are from record c00128_Scaffol.. (original name was: Scaffold_Scf_1152):					
Cluster 5	T1pks	1	19905	-	-
The following clusters are from record c00130_Scaffol.. (original name was: Scaffold_Scf_1154):					

The 66 gene clusters 66 clusters, which include 27 PKSs, 8 NRPS,4 NRPS-PKSs hybrids, 12 for terpene biosynthesis, and 15 other gene clusters. antiSMASH predicted the gene cluster of *M. eutypoides*[3–7].

Figure S2. The sensitivity of the *M. eutypoides* strain to different concentrations of hygromycin B.

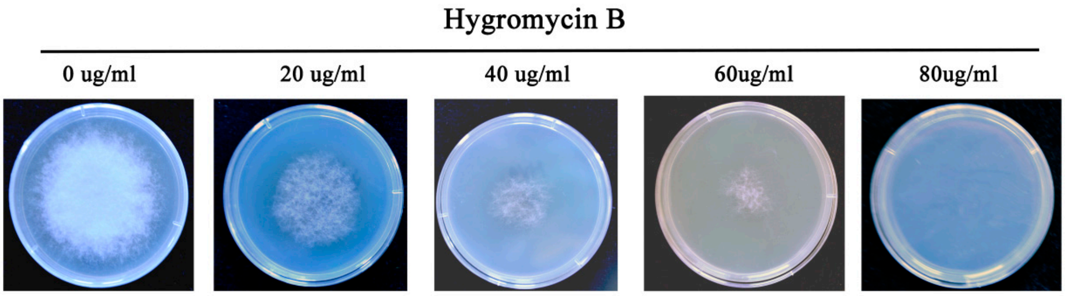

The fungus was grown on the PDA medium supplemented with hygromycin B at 28 °C for 7 days. The concentrations of antibiotics are indicated.

Figure S3. Sequence alignment of Mehat protein

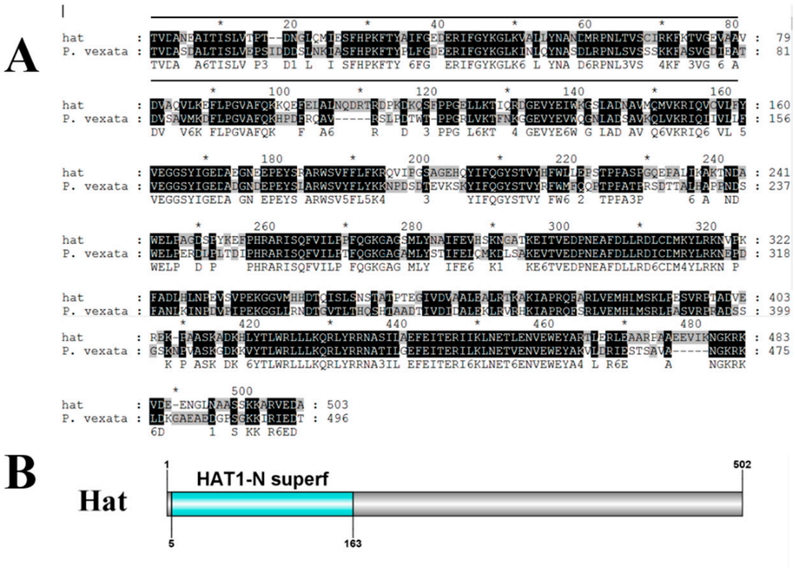

Protein domain alignment (A) and Molecular structure (B) of *hat* and *Pseudomassariella vexata* (Genbank Accession No. ORY69883.1). The conserved regions containing the Hat1-N superfamily domain were labeled with scribe lines, and the comparative analysis software was GeneDoc.

Figure S4. PCR, fluorescence microscopy observation and Western blot of the transformants of *M. eutypoides*.

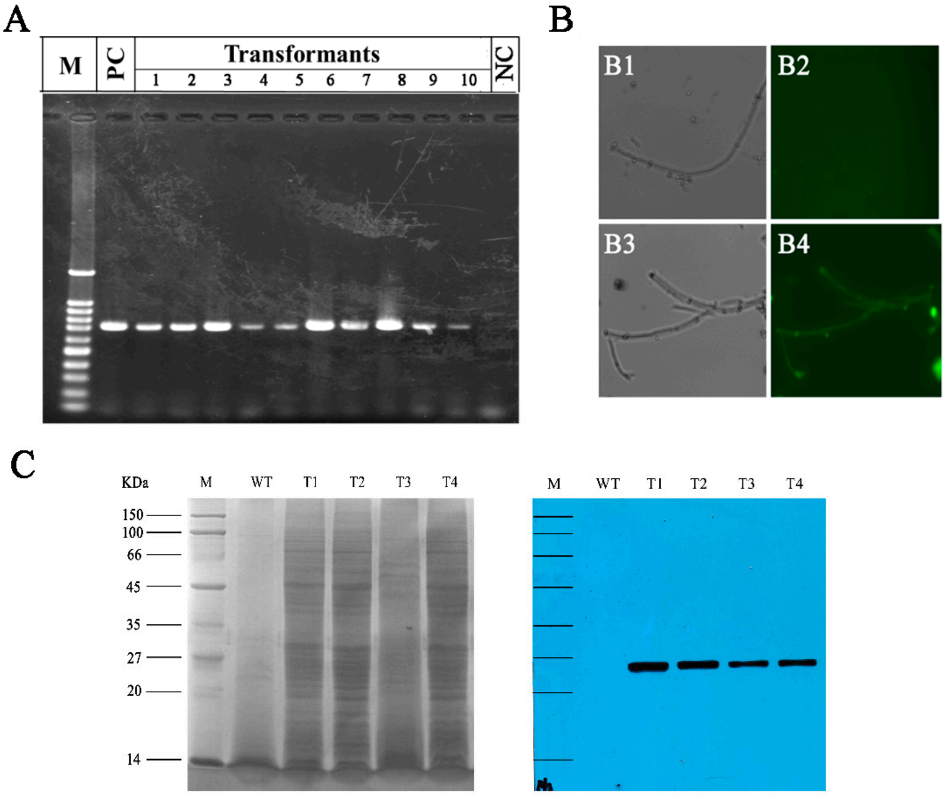

A: PCR analysis of transformants of *M. eutypoides*. The *hyg* gene (858 bp approximate) in ten selected transformants of *M. eutypoides* were amplified by PCR, Transformants: the identified transformants; NC: negative control (wild-type genomic DNA); PC: positive control (pAg1-H3-EGFP plasmid DNA); M: 100 bp ladder. B: Fluorescence microscopy observation of putative transformant mycelia B1: the mycelia of wild type under bright field image; B2: the mycelia of wild type under the fluorescence microscopy; B3: the mycelia of transformant under bright field image; B4: the mycelia of transformant under the fluorescence microscopy. C: Intacellular protein profiles (left panel) and Western Blot. Analysis (right panel) of *M. eutypoides* transformant of day 3 (T1), day 5 (T2), day 7 (T3), day 10 (T4) and wild-type (WT), Molecular size marker (M)

Figure S5. HPLC analysis of all these compounds with standard method.

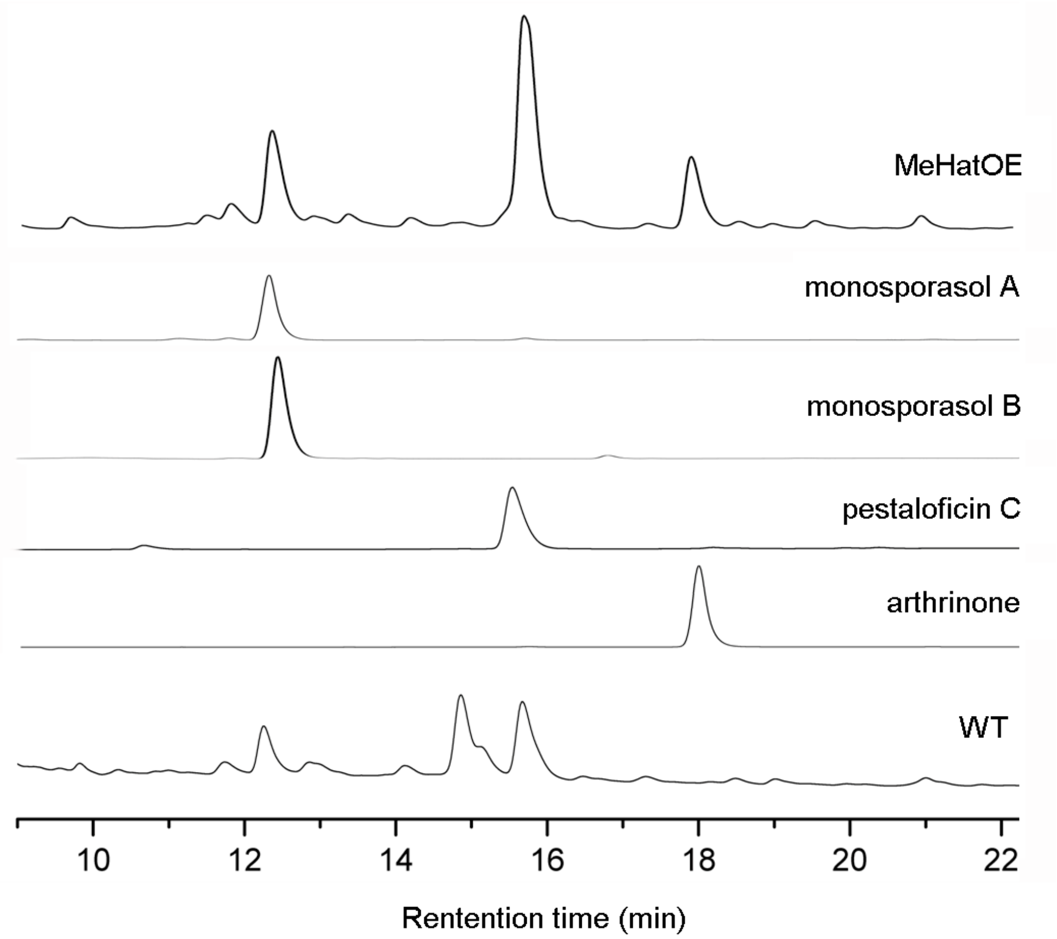

Four compounds of 1: monosporasol A, 2: monosporasol B, 3: pestaloficin C, 4: arthrinone were measured under 254 nm. (Chromatographic conditions: Chromatographic conditions: 0-2 min 60% MeOH in water, 2-25 min 60-100% MeOH/H_2O, 25-30 min 100% MeOH, t = 30 min, 1.0 mL/min, 254 nm.)

Figure S6. IR spectrum of compound **1.**

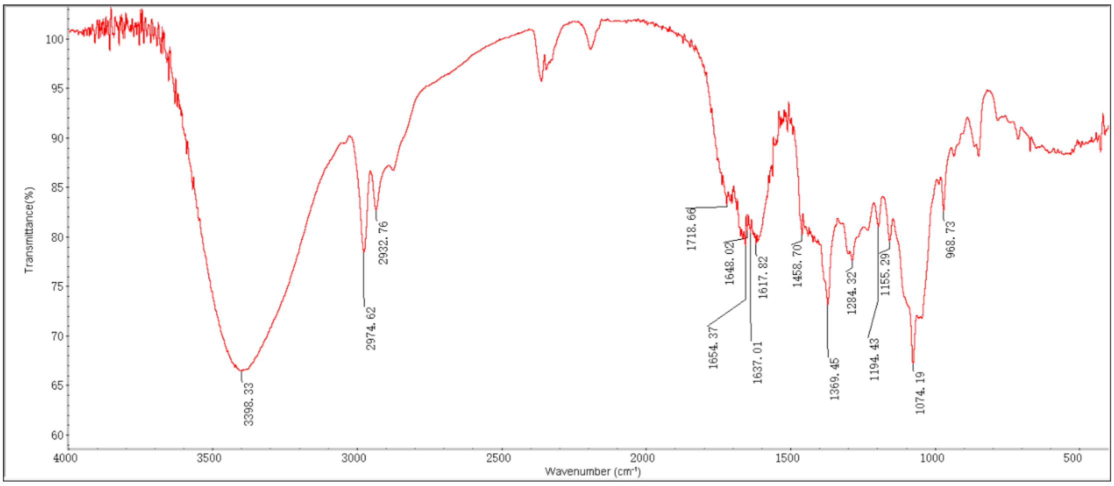

Figure S7. HRESIMS spectrum of compound **1**

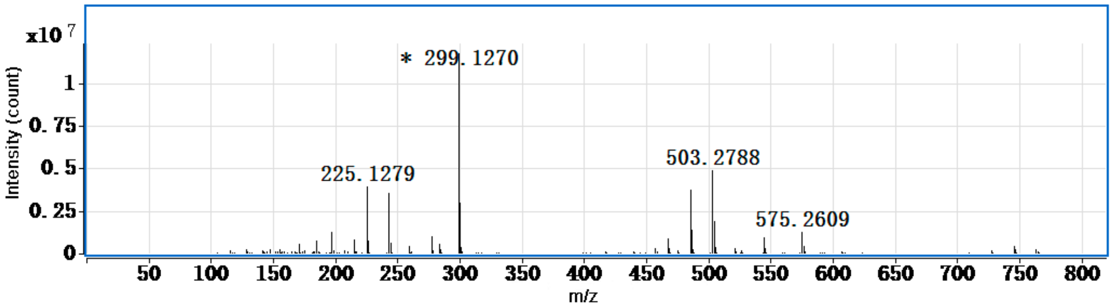

Figure S8. ^{1}H NMR spectrum of compound **1** (600 MHz, Acetone-d_6)

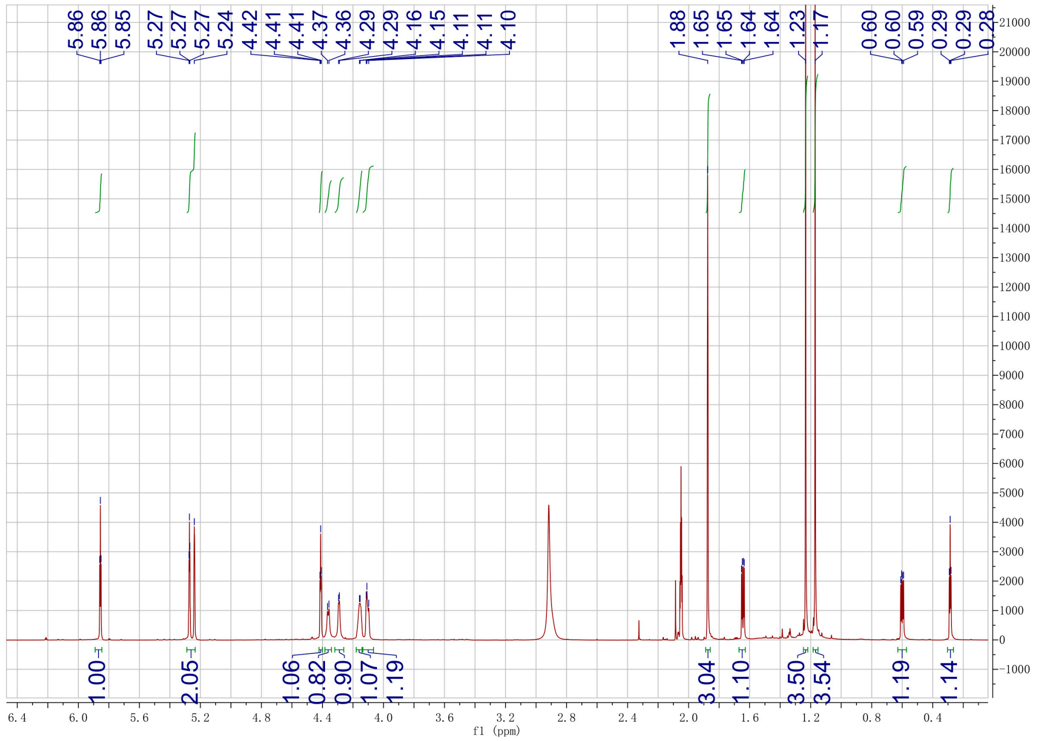

Figure S9. ^{13}C NMR spectrum of compound **1** (150 MHz, Acetone-d_6)

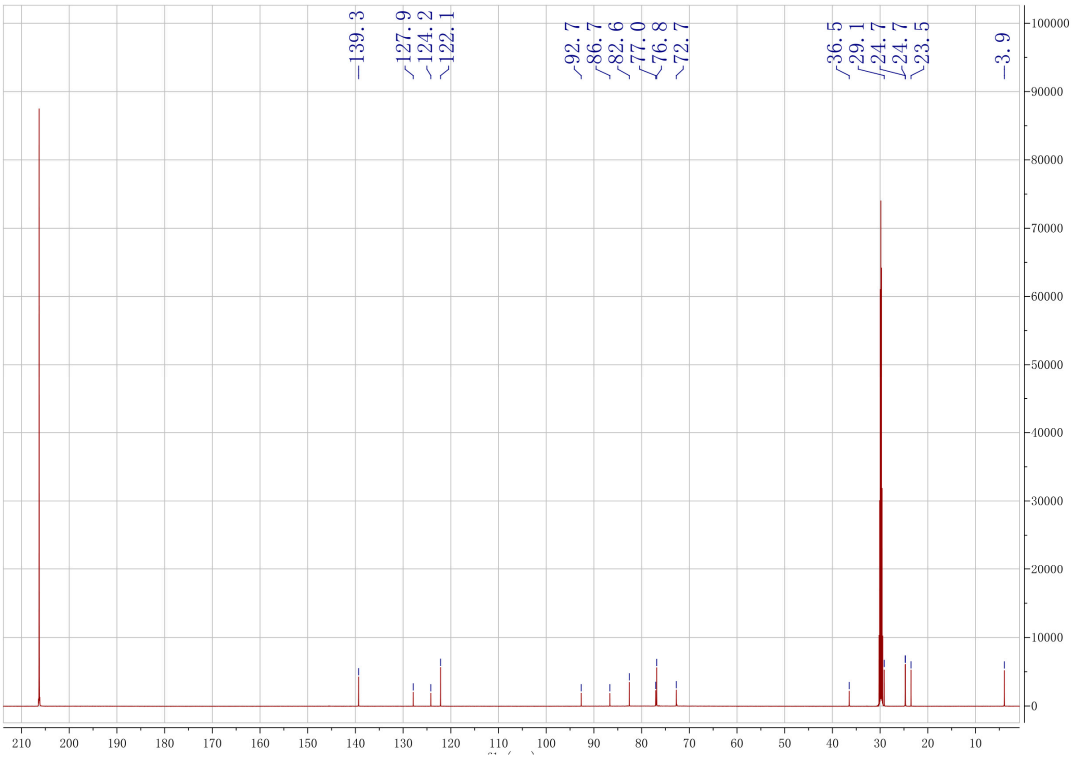

Figure S10. HSQC spectrum of compound **1** (600 MHz, Acetone-d_6)

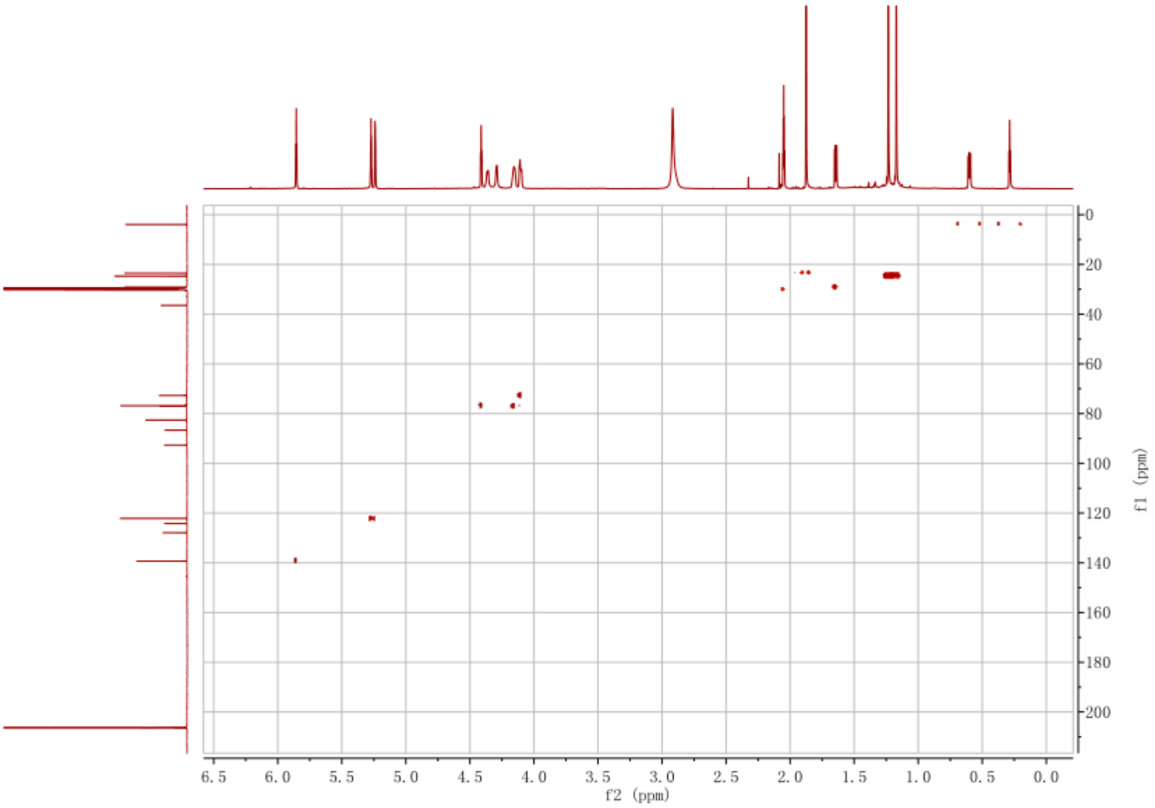

Figure S11. ^{1}H-^{1}H COSY spectrum of compound **1** (600 MHz, Acetone-d_6)

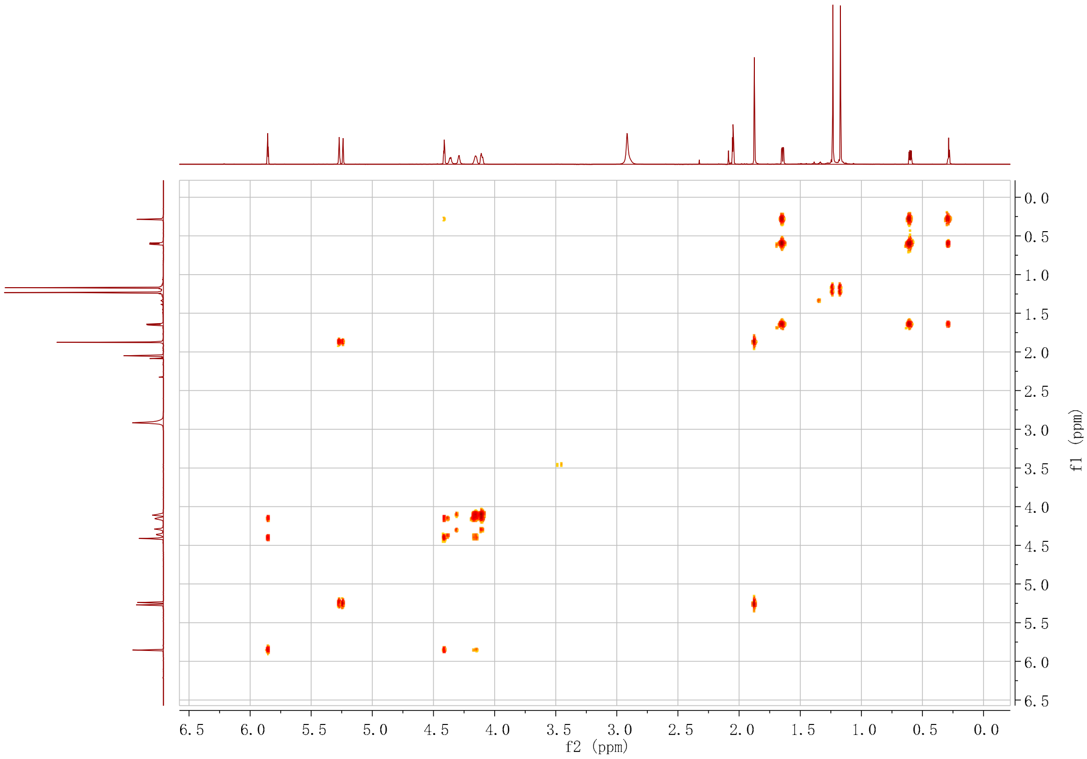

Figure S12. HMBC spectrum of compound **1** (600 MHz, Acetone-d_6)

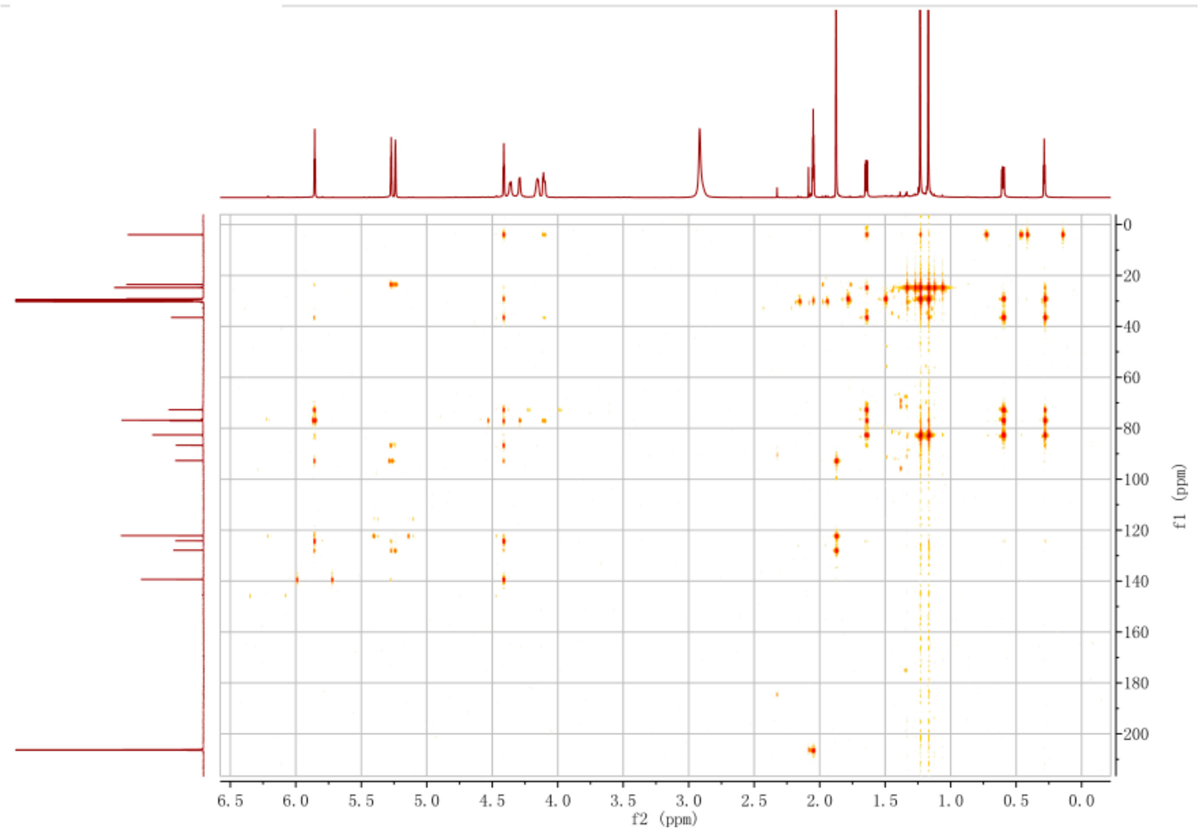

Figure S13. NOESY spectrum of compound **1** (600 MHz, Acetone-d_6)

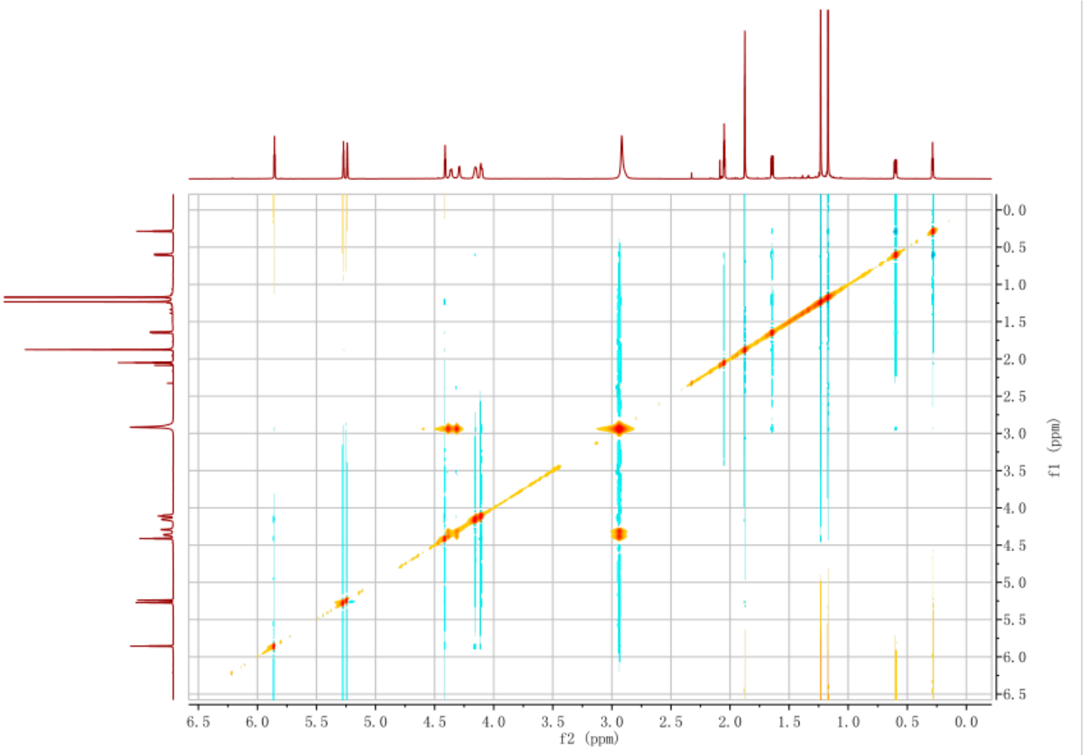

Figure S14. IR spectrum of compound **2**

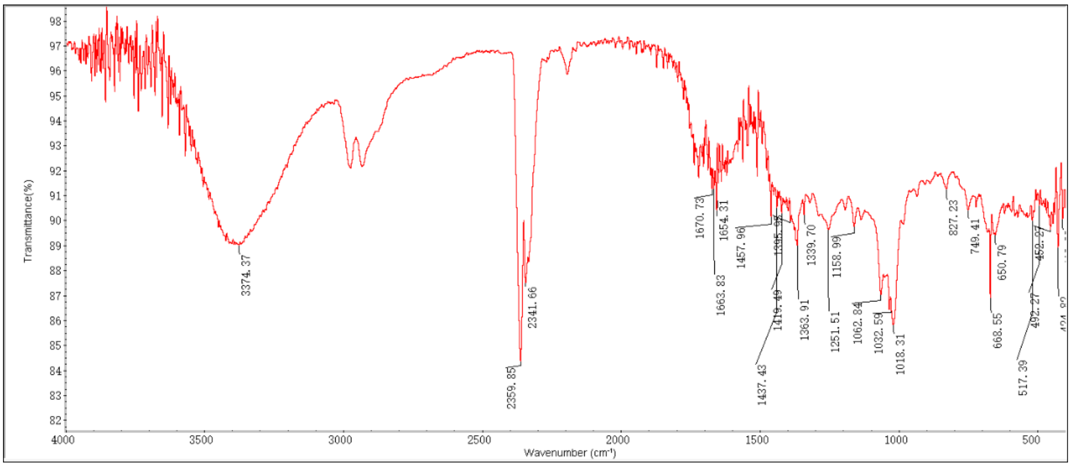

Figure S15. HRESIMS spectrum of compound **2**

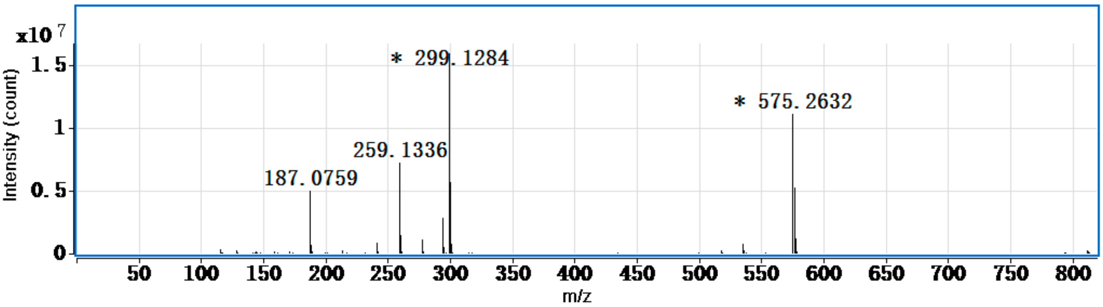

Figure S16. ^{1}H NMR spectrum of compound **2** (600 MHz, Acetone-*d*$_6$)

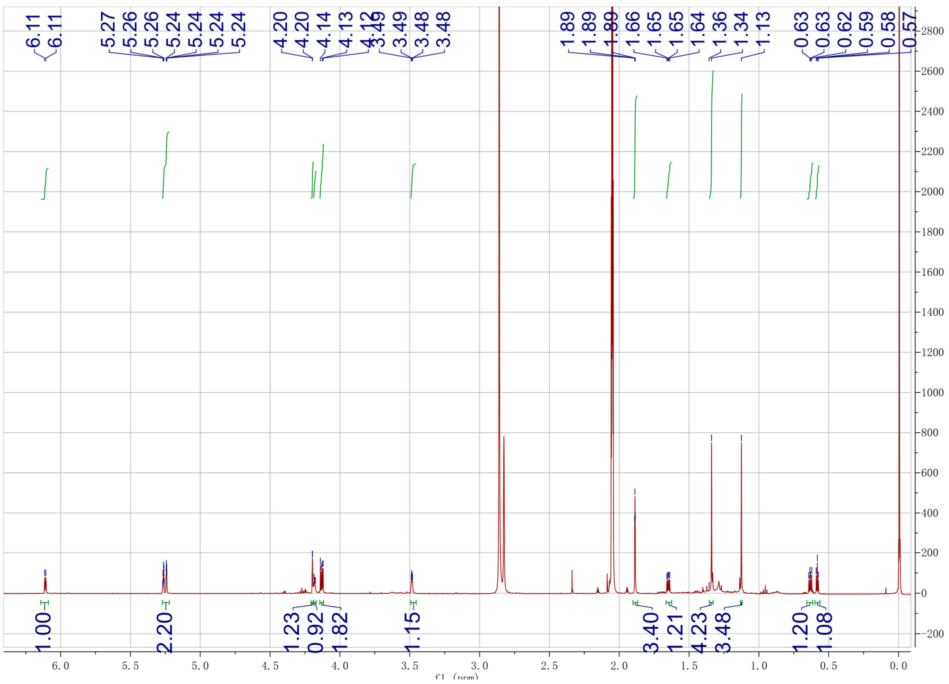

Figure S17. ^{13}C NMR spectrum of compound **2** (150 MHz, Acetone-*d*$_6$)

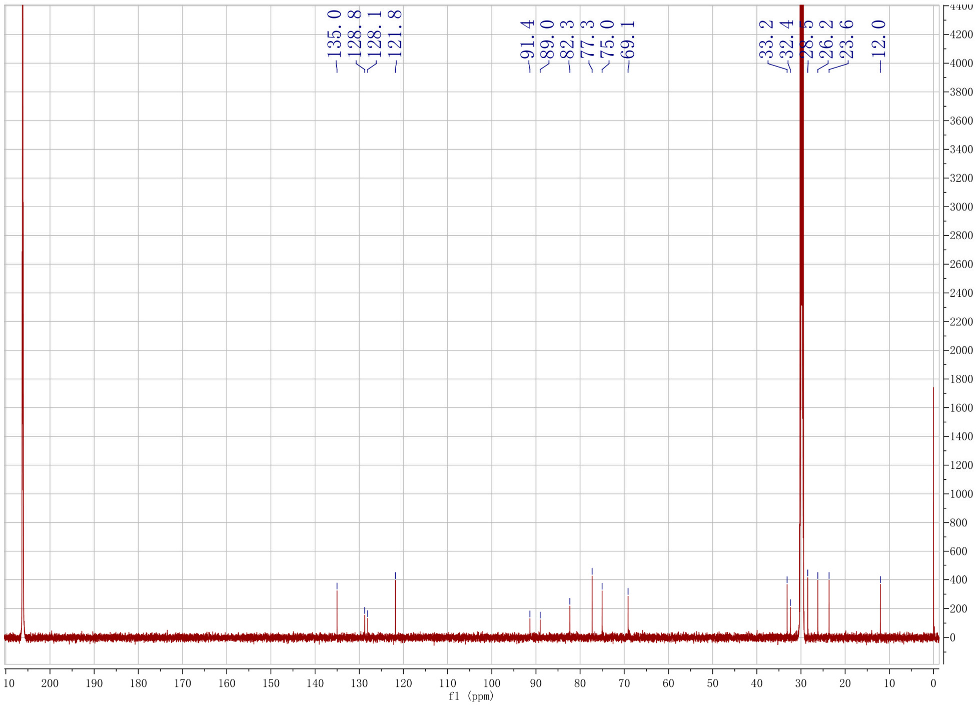

Figure S18. HSQC spectrum of compound **2** (600 MHz, Acetone-d_6)

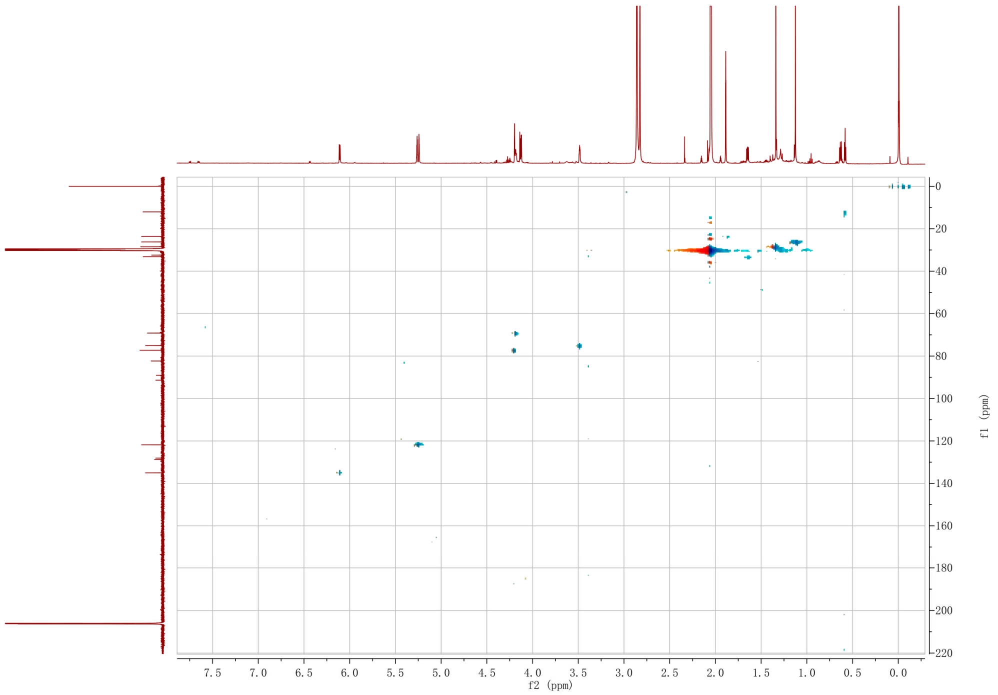

Figure S19. ^{1}H-^{1}H COSY spectrum of compound **2** (600 MHz, Acetone-d_6)

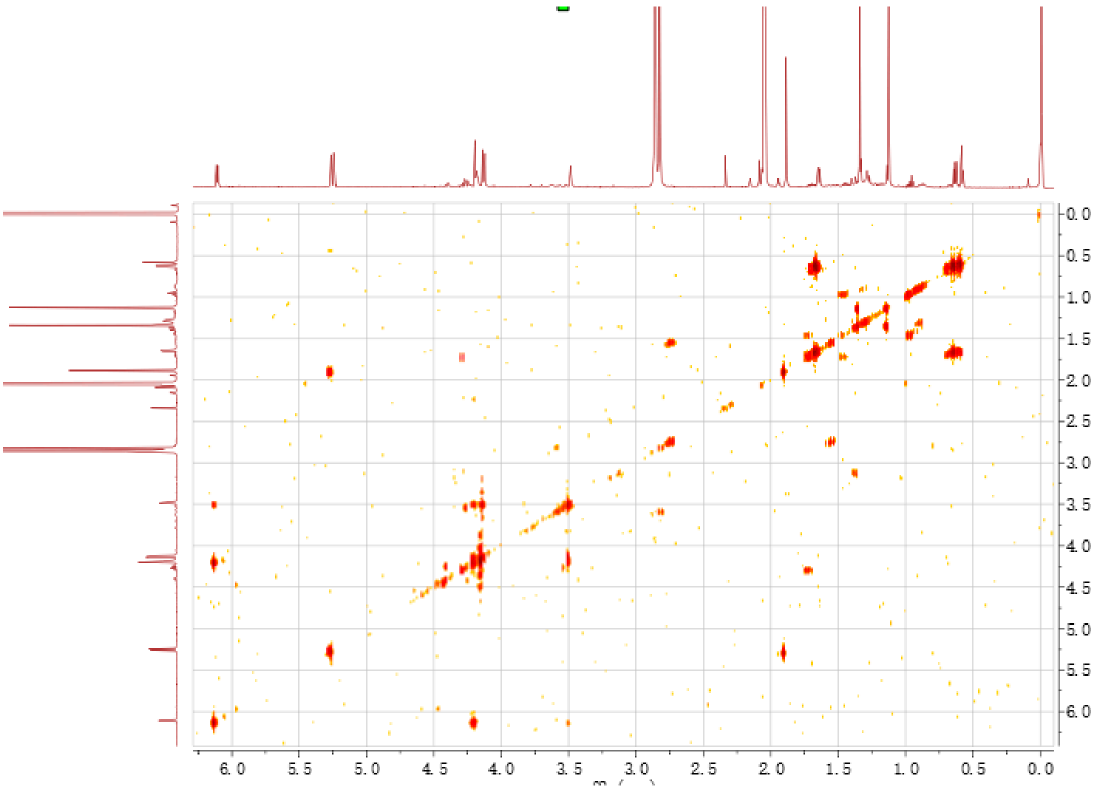

Figure S20. HMBC spectrum of compound **2** (600 MHz, Acetone-d_6)

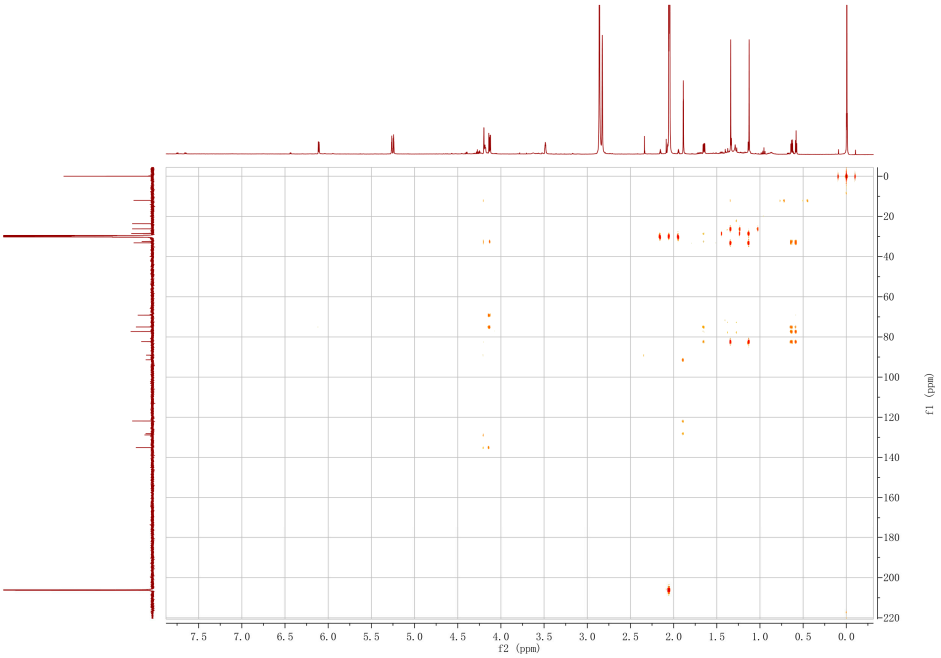

Figure S21. NOESY spectrum of compound **2** (600 MHz, Acetone-d_6)

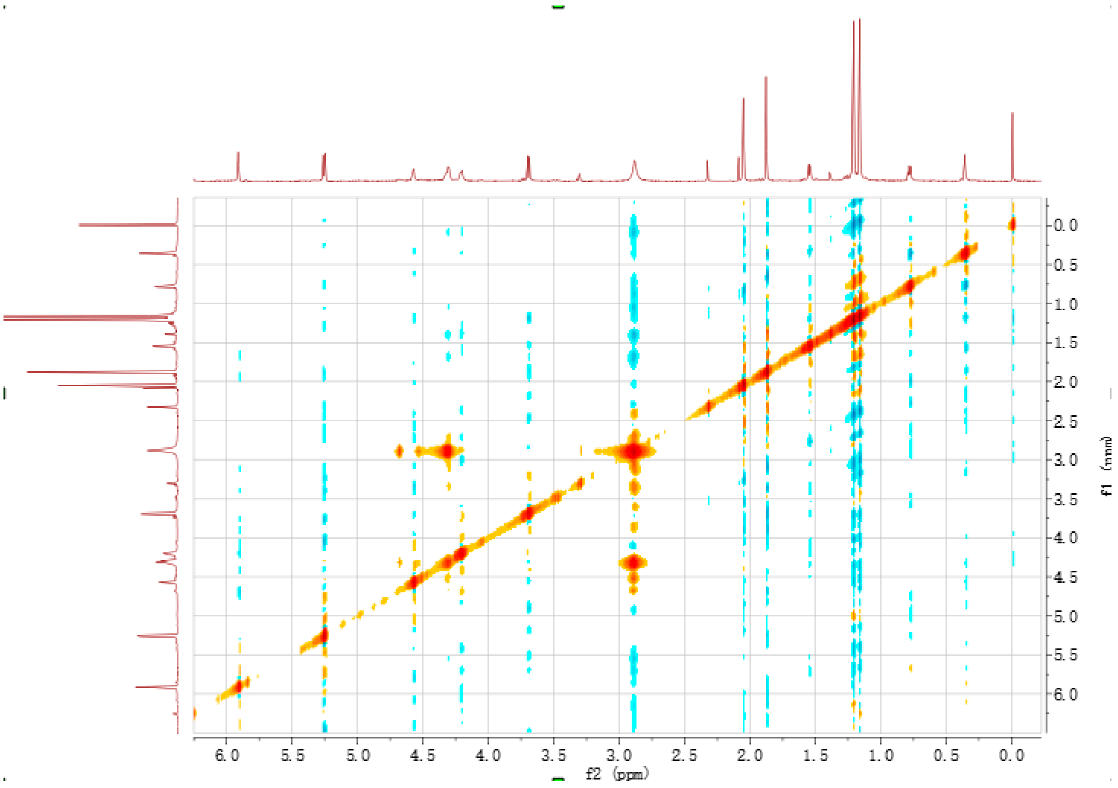

Figure S22. Five Conformations of **1a** were obtained after the Optimization

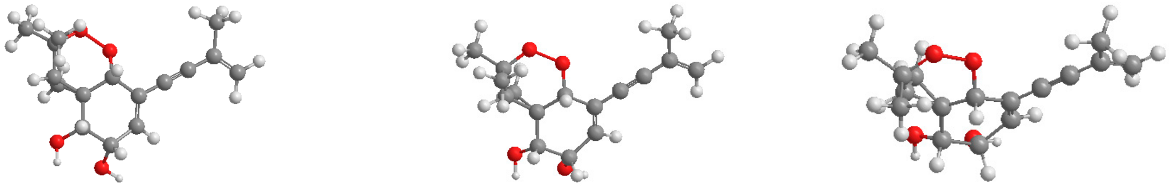

1aC1(21.06) 1aC2(21.11) 1aC3(17.54)

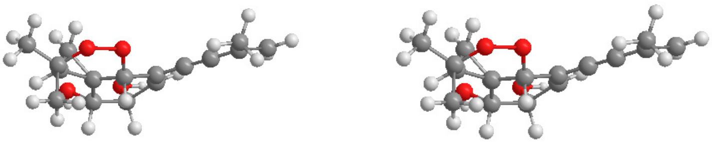

1aC4(22.28) 1aC5(18.02)

Figure S23. Twenty Conformations of **2a** were obtained after the Optimization

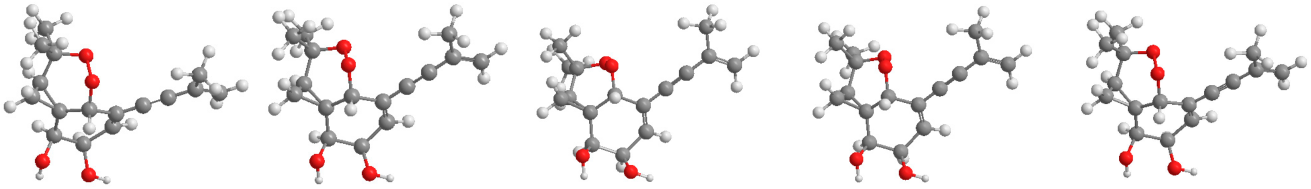

2aC1(4.03) 2aC2(3.56) 2aC3 (3.45) 2aC4(4.03) 2aC5 (4.14)

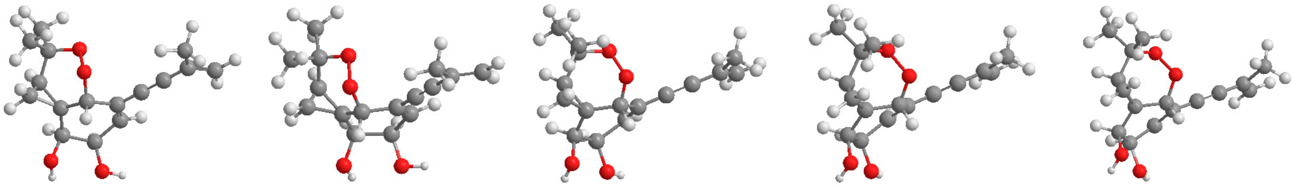

2aC6(3.50) 2aC7(7.78) 2aC8(6.29) 2aC9(7.76) 2aC10(7.56)

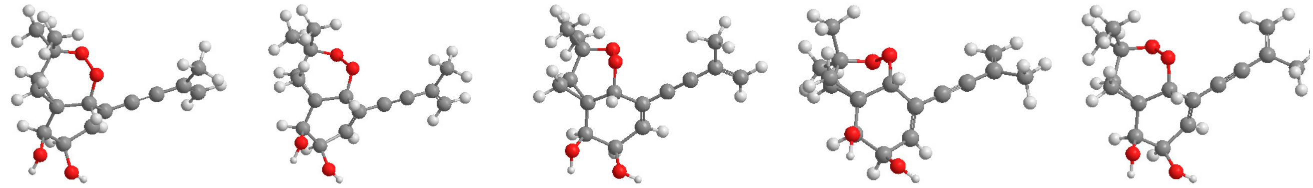

2aC11(6.43) 2aC12(7.79) 2aC13(6.20) 2aC14(6.11) 2aC15(3.84)

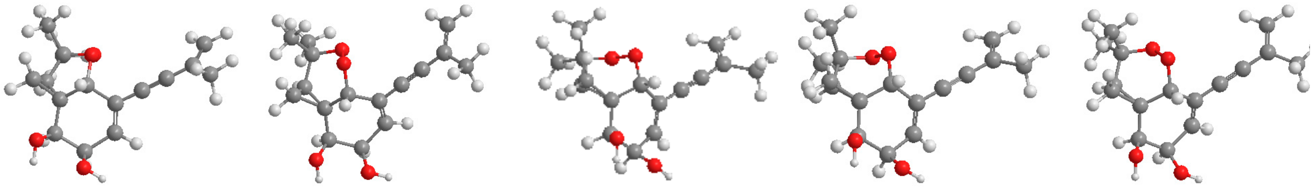

2aC16(3.87) 2aC17(3.19) 2aC18(3.25) 2aC19(3.97) 2aC20(3.35)

References

1. Khang, C.H.; Park, S.Y.; Rho, H.S.; Lee, Y.H.; Kang, S. Filamentous Fungi (Magnaporthe grisea and Fusarium oxysporum). *Methods in Molecular Biology* **2006**, 344, 403-420.

2. Yanling, W.; Pengjie, H.; Erwei, L.; Xingzhong, L.; Yongsheng, C.; Gang, L. Genetic transformation of the fungus Gliocladium sp. mediated by *Agrobacterium tumefaciens*. *Acta Microbiologica Sinica* **2013**, 53, 1233-1239.
3. Blin, K.; Wolf, T.; Chevrette, M.G.; Lu, X.; Schwalen, C.J.; Kautsar, S.A.; Suarez Duran, H.G.; De, l.S., Emmanuel L. C; Kim, H.U.; Nave, M.J.N.A.R. antiSMASH 4.0—improvements in chemistry prediction and gene cluster boundary identification. **2017**.
4. Weber, T.; Kai, B.; Duddela, S.; Krug, D.; Kim, H.U.; Bruccoleri, R.; Sang, Y.L.; Fischbach, M.A.; Müller, R.; Wohlleben, W.J.N.A.R. antiSMASH 3.0—a comprehensive resource for the genome mining of biosynthetic gene clusters. **2015**, 43, W237.
5. Kai, B.; Medema, M.H.; Daniyal, K.; Fischbach, M.A.; Rainer, B.; Eriko, T.; Tilmann, W.J.N.A.R. antiSMASH 2.0--a versatile platform for genome mining of secondary metabolite producers. **2013**, 41, W204.
6. Medema, M.H.; Kai, B.; Peter, C.; Victor, D.J.; Piotr, Z.; Fischbach, M.A.; Tilmann, W.; Eriko, T.; Rainer, B.J.N.A.R. antiSMASH: rapid identification, annotation and analysis of secondary metabolite biosynthesis gene clusters in bacterial and fungal genome sequences. **2011**, 39, W339.
7. Blin, K.; Shaw, S.; Steinke, K.; Villebro, R.; Ziemert, N.; Lee, S.Y.; Medema, M.H.; Weber, T.J.N.A.R. antiSMASH 5.0: updates to the secondary metabolite genome mining pipeline.
